# Higher Education and Race Suicide

**Published:** 1915-07

**Authors:** 

**Affiliations:** San Antonio


### Higher Education and Race Suicide.

Apropos to the above startling conditions, quoting from the same authority, attention is called to the effect of higher education on reproduction. If statistics prove anything, the experience of our leading schools for the higher education of women uncovers a condition that is menacing. These facts are not brought forth to disparage higher education for women, but to emphasize the tendency of modern customs to complicate social conditions.

Perhaps it is not the effect of higher education so much as it is the growing spirit of financial independence. Education develops a higher appreciation of marriage and the responsibility of parenthood, and, in this way, ought to be a strong eugenic agency. While it results eventually in a much lower birth rate, it must be borne in mind that a lower mortality rate follows the lower birth rate, and in the end increases the population, providing the birth rate does not fall below the necessary line to reproduce a number sufficient to maintain a constant ratio.

But as to the effect higher education has on reproduction itself should and must be considered. The following statistics are indeed interesting, and should receive serious thought: Of the graduates of Mt. Holyoke, 41.9 per cent married; 32.8 per cent of those of Bryn Mawr, and 31 per cent of Wellesley. The Mt. Holyoke graduates have averaged, in reproduction, .8 of a child; Vassar, 1.74, and Wellesley 1.17.

Conceding this conclusion to be correct, the fact emphasizes the perilous condition that now confronts the race in the better classes limiting their offspring, while the poorer and less fit classes are increasing theirs in overwhelming proportions. Especially is this true of the more or less degenerate classes and the less desirable immigrants coming to this country. The effect of this is a constant deterioration of our racial stock which can lead but to one end.

